# Post-Discharge Effects and Parents’ Opinions of Intranasal Fentanyl with Oral Midazolam Sedation in Pediatric Dental Patients: A Cross-Sectional Study

**DOI:** 10.3390/children9020142

**Published:** 2022-01-22

**Authors:** Roaa I. Alhaidari, Maha A. AlSarheed

**Affiliations:** Department of Pediatric Dentistry and Orthodontics, College of Dentistry, King Saud University, Riyadh 11545, Saudi Arabia; malsarheed@ksu.edu.sa

**Keywords:** pediatrics, moderate sedation, midazolam, fentanyl, questionnaire

## Abstract

The aim of this study was to evaluate the post-discharge effects of oral midazolam with intranasal fentanyl sedation in pediatric patients who had dental treatment and to evaluate parents’ preference regarding sedation visits. Methods: A total of 32 uncooperative healthy pediatric patients aged 3–6 years old who met the inclusion criteria were included. In the first visit, one group received oral midazolam (0.7 mg/kg) with intranasal fentanyl (1 μg/kg) sedation (M/F) and the other group received oral midazolam with intranasal placebo (M), and in the second visit each group received the other type of sedation in a cross-over type. In this cross-sectional study, a post-discharge phone-call questionnaire was carried out 24 h after both sedation visits with the parents to evaluate the children’s behavior, function, balance, eating pattern, sleeping pattern, vomiting incidents, and any possible side effects, as well as parents’ satisfaction and preference. The Wilcoxon signed-rank test was used to analyze the categorical variables, and the Chi-square test was performed to analyze the parents’ preference. Result: A total of 32 parents responded to the phone-call questionnaire after 64 sedation visits. All of them were mothers. There was no statistically significant difference between the two groups with respect to recovery to normal function and balance, behavior, incidents of fever, vomiting, sleep disturbance, oversleeping, and adverse behavioral changes (*p* > 0.05). Children required a significantly longer amount of time until the first meal after M/F sedation (*p* = 0.04). No significant difference was found between parents’ preferences regarding the sedation visits (*p* > 0.05). Conclusion: Intranasal fentanyl added to oral midazolam sedation could have an effect on post-discharge adverse behavioral changes, prolonged sleeping, and prolonged recovery time. Children sedated with midazolam/fentanyl required a longer amount of time until the first meal. Vomiting and fever occurred similarly in both sedation regimens with a low incidence. There was no difference in parents’ preferences regarding the two sedation regimens.

## 1. Introduction

The delivery of diagnostic or therapeutic procedures to young, uncooperative, and anxious pediatric patients poses numerous challenges to the operator [1]. Conventional non-pharmacological behavior management along with local anesthesia is commonly utilized with most pediatric patients in dental clinics [2]. However, for some patients with behavioral management problems such as fear and anxiety, sedation is needed to deliver the needed dental treatment safely [3]. Moderate sedation is utilized to provide the needed dental treatment to anxious children to avoid psychological distress and poor compliance during or after treatment, as well as the possibility of cancelation during the treatment [4].

Sedative drugs can be administered through different routes, such as the intravenous, intramuscular, oral, and intranasal routes [5]. In dentistry, the oral route is the most common technique used as it is convenient, tolerable, and less distressing to the child [6,7,8].

Midazolam is a common sedative drug delivered to pediatric patients orally to reduce anxiety in the dental clinic [6,9,10]. Midazolam is a benzodiazepine derivative that has sedative, anxiolysis, and anterograde amnesia properties with a wide margin of safety [11,12,13].

Previous studies found moderate evidence indicating that oral midazolam sedation is effective for pediatric patients during dental treatment [3]. However, oral midazolam has a relatively low bioavailability because of the hepatic first-pass metabolism effect [14,15]. Some studies found that combining oral midazolam with other sedative drugs produces better sedation and behavior in pediatric patients undergoing dental treatment [6,16].

Notably, there has been an increased interest in drugs with both sedative and analgesic properties, such as dexmedetomidine, ketamine, and fentanyl [17,18].

Fentanyl is a potent and highly selective μ-opioid agonist that has a relatively high margin of safety [19,20,21]. Combining midazolam with fentanyl in pediatric sedation may result in a synergistic effect; however, the side effects are dose-dependent [22]. Previous studies found that midazolam with fentanyl sedation delivered by the intravenous and intranasal routes produced safe and effective sedation [18,23,24,25]. However, some studies found that combining 0.5 mg/kg oral midazolam with 3 μg/kg submucosal fentanyl or with 5–10 μg/kg oral transmucosal fentanyl produced more side effects, which could be attributed to the high doses of fentanyl used [26,27].

Pediatric dental sedation has an excellent safety record [8,11,28,29]. Nevertheless, complications can still occur [30]. The possible adverse effects during and after sedation could be reduced, but not completely eliminated, by a meticulous preoperative review of the patient’s medical status, consideration of how the sedation might be affected by these conditions, and by following the discharge criteria listed in the American Academy of Pediatric Dentistry (AAPD) guidelines before discharging the patient [4]. Premature discharge of patients having received long plasma half-life sedative drugs, such as chloral hydrate, pentobarbital, and promazine, can result in post-discharge adverse effects [31,32]. Some possible adverse effects that could happen after oral sedation are nausea, vomiting, prolonged sleeping, irritability, difficulty in speaking or walking, changes in activity or behavior, and fever [33,34,35,36,37].

Few studies have been conducted to investigate the post-discharge adverse effects of some sedative drugs in pediatric patients [9,32,33,34,35,36,37]. To our knowledge, there seem to be no investigations in the pediatric literature assessing the post-discharge adverse effects of oral midazolam with intranasal fentanyl sedation. Therefore, the objective of the present study was to compare the post-discharge effect of oral midazolam with intranasal fentanyl against sedation using oral midazolam only. Additionally, the parents’ preference regarding the two sedative regimens investigated was evaluated.

## 2. Materials and Methods

### 2.1. Sample Selection and Study Protocol

This cross-sectional study was approved by the Institutional Review Board (IRB No. E-19-3953) and the Ethics Committee of the College of Dentistry Research Center “CDRC No. PR 0106” of King Khaled Medical City at King Saud University in Riyadh, Saudi Arabia. This study is registered in the International Standard Randomized Controlled Trial Number registry under study ID ISRCTN 13661311. The ethical principles proposed by the World Medical Association Declaration of Helsinki were followed throughout this study. The present study is the second part of a previously conducted clinical trial. The first part was a cross-over randomized clinical trial that evaluated the sedative effect and safety of oral midazolam with intranasal fentanyl versus oral midazolam with intranasal placebo in pediatric patients during dental treatment. Children were randomly selected from the sedation waiting list in the pediatric dental clinics of the Dental University Hospital of King Khaled Medical City at King Saud University for dental treatment under moderate sedation after obtaining written consent from parents for their children to participate in the study according to the following inclusion criteria: 3–6 years old, American Society of Anesthesiologists (ASA) physical status I [38], Frankl behaviour rating scale of 1 or 2 [39], Mallampati score of class I or II [40], Brodsky tonsillar size scoring of 0, 1 or 2 [41], children within the normal range of weight [42], children who needed two sedation visits for the completion of the dental treatment (children who needed more than two sedation visits were referred to be treated under sedation with another dentist and were not included in the study), and children who needed a comparable dental treatment (restorations, pulp treatments, crowns, or extractions) on both sides of the same jaw. Exclusion criteria were children with learning difficulties or mental disabilities, active upper respiratory tract infection, any history of a recent cough or cold (less than two weeks), children with a known allergy or hypersensitive reaction to either midazolam or fentanyl, children at risk of airway obstruction (obstructive sleep apnea or a craniofacial syndrome), children with any intranasal pathology or congenital anomaly, children with a previous history of moderate sedation (to eliminate recall bias), and children whose parents refused to allow them to participate. On the day of the sedation, the child was examined for medical clearance, and their weight and physiological parameters (blood pressure (BP), heart rate (HR), and oxygen saturation (SpO_2_)) were recorded. The patients were randomly selected to receive either oral midazolam with intranasal fentanyl (M/F) in one visit or oral midazolam with intranasal saline as placebo (M) in the other visit using a random number table. The randomization numbers were concealed in opaque sealed envelopes that were opened after the parents gave consent on the day of sedation by a trained nurse who prepared the sedative drugs to be delivered to the patient. Then, two blinded, trained dental operators delivered the sedative drugs to the child in the presence of the parents and under the supervision of the anesthesiologist. The doses of midazolam and fentanyl were calculated according to the weight of the child: 0.7 mg/kg for the midazolam [43] (lab-formed midazolam, 2 mg/mL [44,45]) and 1 μg/kg for the parenteral fentanyl [21,46] (fentanyl, 100 μg/2 mL). Midazolam syrup was delivered orally, and after 10 minutes, either fentanyl or normal saline was delivered intranasally through an atomizer with half of the amount in the right nostril and the other half in the left nostril to maximize the absorption. After this, when the child was sedated, he/she was transferred immediately to the dental chair, and the parents were asked to wait in the waiting area. All patients were stabilized using a size-appropriate papoose board to guard their safety, and then topical anesthesia was applied at the site of the injection followed by local anesthesia as infiltration for the upper arch or inferior alveolar nerve block (IANB) for the lower arch. After this, a mouth prop was placed in the area opposite the working site, and a rubber dam was applied; then, dental treatment was delivered to the patients by two blinded dental operators who had a comparable level of clinical experience. The required dental treatment was any of the following: stainless steel crown, glass ionomer, composite resin, and preventive resin restoration (type of conservative adhesive restoration), or extraction if indicated. The patient’s physiological parameters (BP, HR, and SpO_2_) were monitored throughout the sedation and in the recovery room by a trained dental assistant until the child was discharged. Any decrease of 20% of the BP or HR baseline was documented, as well as any reduction of SpO_2_ below 92%. Flumazenil and naloxone were prepared and dosed for each sedation visit according to the patient’s weight and administration characteristics to be used if necessary; however, these were not needed during our study. The child remained in the recovery room with his/her parents and was then discharged when he/she fulfilled the AAPD discharge criteria [4]. Post-sedation instructions in both verbal and written forms were given to the parents before the child’s discharge [47]. The same operator treated the same child for both sedation visits. The period between the first and the second visit was more than two weeks and less than four weeks. The blinded principal investigator called the same blinded parent 24 h after both sedation visits to conduct the questionnaire [9] (Appendix A). The parents were asked about their sedation visit preferences after the second visit only. After completing the questionnaires for the whole sample, the sedation groups were revealed by the investigator for statistical analysis.

### 2.2. Questionnaire

A previously published phone-call questionnaire was utilized after obtaining written permission from the author [9]. The questionnaire consists of 10 questions divided into two sections: The first part, that was asked after both sedation visits, consists of questions regarding possible post-discharge adverse effects, which are: vomiting frequency, time until the child had their first meal and functioned normally, time until the child regained balance and normal vision, behavior status, sleeping pattern, parents’ satisfaction, and any complications/side effects encountered. Parents were asked if vomiting occurred or not; in the cases where vomiting had occurred, more questions regarding the quantity and time in relation to the sedation time were asked. Moreover, parents were asked about the time needed to regain balance, normal vision, and eat and function normally, then the answer was marked accordingly by the investigator in the multiple time choices of the questionnaire. However, the preconstructed answers to the questions regarding the behavior status of the child, sleeping pattern, and parents’ satisfaction were verbally delivered to the parents, and they chose the appropriate answers according to their child. The complications/side effects question was asked as an open-ended question of the parents. The second part was asked after the second sedation visit only and it concerned parents’ preferences regarding any of the sedation visits. They were asked if their child had to do an extra sedation visit, which one of the previous two sedation visits they would choose.

### 2.3. Validity and Reliability of the Questionnaire

The questionnaire was originally published in English; thus, it was translated into Arabic language by a certified translation agency and then into the English language to test the reliability of the translation. The content validity of the questionnaire was assessed by two experts in pediatric sedation who were asked about the appropriateness and phrasing of each question. The face validity was assessed by asking 10 mothers not involved in the study to evaluate their understanding of the questions. The reliability test was performed on 10 mothers not involved in the study, and the result was assessed using Cronbach’s alpha (*α*), which resulted with an average of *α* = 0.886, indicating that the questionnaire showed good repeatability.

### 2.4. Sample Size

The sample size was calculated based on the assumptions of an alpha of 0.05, a power of 0.90 (90%), and an estimated effect size of 0.5. Based on these assumptions, the sample size was 30. After estimating a 20% drop-out, the desired sample size was 36.

The data analysis was conducted using the SPSS program (IBM Inc., Chicago, IL, USA). Descriptive statistics and Wilcoxon’s signed-rank test were used to describe and analyze the categorical variables. The Chi-square test and Fisher’s exact test, where applicable, were performed to analyze the behavior scores during and after the sedation visit as well as the parents’ preference. All statistical analyses were set with a significance level of *p* < 0.05. 

## 3. Results

A total of 32 parents answered the questionnaire for 64 sedation visits with a response rate of 100%. All of them were the children’s mothers. The demographic data of the children are shown in Table 1.

The association between the behavior of pediatric patients during the sedation visit assessed in the first part of the study and the behavior after discharge assessed through the post-discharge questionnaire was evaluated.

In the M/F group, there was a significant negative correlation between behavior during sedation and behavior after discharge (*r* = −0.419, *p* = 0.017). A total of 18 (56.3%) children had calm behavior or were easily calmed during sedation; of these, seven (38.9%) children continued with a normal or relaxed behavior at home, while the rest became moderately agitated, and one child (5.6%) became very agitated at home. The remaining 14 children (43.7%) were either moderately or very agitated during sedation; however, 11 children had normal behavior or were even relaxed at home, and the remaining three children continued with moderate or very agitated behavior at home.

In the M group, there was a negative correlation between behavior during sedation and behavior after discharge, but it was not statistically significant (*r* = −0.134, *p* = 0.465). Only 13 children (40.6%) had calm or easily calmed behavior during sedation; of these, eight children (61.5%) continued with normal or relaxed behavior at home, while the rest became moderately or very agitated. The remaining 19 children (59.4%) had moderate or very agitated behavior during sedation, and 13 of them had normal behavior or became relaxed at home, while the rest continued with moderate or very agitated behavior.

A total of three children vomited after the sedation visit (two children (6.3%) in the M/F group and one child (3.1%) in the M group). All three children vomited the first meal they had after sedation. Two children in the M/F group vomited 2–4 h after sedation, and one child in the M group vomited after more than six h (Table 2).

When the parents were asked when the child ate after sedation, the children from the M/F group required a longer time to begin eating compared to children from group M, and this was statistically significant (*p* = 0.04) (Table 2 and Table 3).

When the parents were asked when the child returned to functioning normally, 84.4% of the children in the M/F group needed 4–6 h or more after sedation, while in the M group, the children started to function normally earlier: 37.5% after 2–4 h or less and 21.9% after 4–6 h, whereas 40.6% required more than 6 h (Table 2).

In the M/F group, the children required a longer time to regain balance than those in the M group: 26 children (81.3%) in the M/F group needed 4–6 h or more to regain balance, while 20 children (62.6%) in the M group required 4–6 h or more to regain balance, and the rest needed less time in both groups (Table 2).

In regard to behavior during the day of sedation, 18 children (56.3%) in the M/F group had either normal or relaxed behavior compared to 21 children (65.6%) in the M group. The rest had aggressive or very aggressive behavior (Table 2).

More than half of the children had normal sleep on the day of sedation for both the M/F and M group (59.3% and 62.5%, respectively), while the rest of the children had some changes in their sleeping pattern, such as an increase or decrease in sleeping hours or nightmares (28.1%, 6.3%, and 6.3%, respectively, for the M/F group and 18.7%, 9.4%, and 9.4%, respectively, for the M group) (Table 2).

Side effects occurred in six children (three children from the M/F group (9.4%) and three children from M group (9.4%)). The side effects after M/F sedation were fever (two children, 6.3%) and diarrhea (one child, 3.1%), while the side effect after M sedation was fever (three children, 9.4%). None of the children had abnormal, double, or blurred vision in either sedation regimen (Table 2). 

Three parents (9.4%) answered that they were very unsatisfied or unsatisfied after M/F sedation, while the rest were neutral (15.6%) or satisfied and very satisfied (75%). In the M group, six parents (18.7%) were very unsatisfied or unsatisfied, while four parents (12.5%) were neutral, and 22 parents (68.8%) were satisfied or very satisfied (Table 2).

**Figure 1:**
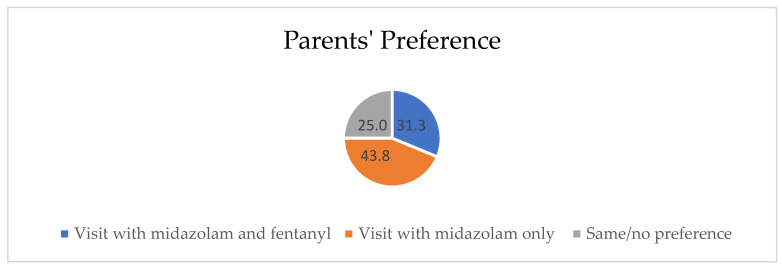
Parents’ preference regarding sedation visits.

In regard to parents’ preference regarding the sedation visits, there was no statistically significant difference between preference regarding the two sedative regimens investigated in this study (Chi-square *p* = 0.417) (Figure 1).

## 4. Discussion

Few studies have been conducted that investigate the post-discharge sedation adverse effects of different combinations of sedative drugs in pediatric patients [9,32,33,34,35,36,37]. Different combinations of oral sedative drugs have produced different post-discharge adverse effects; hence, the provider must select the most appropriate combination of drugs that is suitable to the child, and the parents must be asked to observe the child until he/she has completely returned to normal function [36,37].

In the present study, we found that both sedation regimens produced a comparable level of post-discharge physiological and behavioral outcomes. Adverse effects such as fever, vomiting, adverse behavioral changes, sleep disturbance, and prolonged sleeping occurred in both groups, with no complications reported. In the first two of these adverse effects, a low frequency was recorded for both groups.

In the present study, adding fentanyl to midazolam did not affect the post-discharge vomiting frequency; moreover, the prevalence of vomiting was low for both groups. This study results are consistent with those of previous studies that reported a low prevalence of post-discharge vomiting [33,48,49]. McQueen et al. found that midazolam with fentanyl sedation had a low prevalence of 13% compared to midazolam with ketamine, which had a prevalence of 20% [48]. Another study reported a post-discharge vomiting prevalence of 7% with IV fentanyl/midazolam compared to 15% with IV ketamine/midazolam [49]. However, Martinez et al. reported no vomiting incidents with either a triple combination of chloral hydrate, meperidine, and hydroxyzine or midazolam-only sedation; all were delivered orally to the children for dental treatment [33].

Adverse behavioral effects, although not statistically significant, were more frequently observed in midazolam/fentanyl sedation. Previous studies found a low prevalence of post-discharge adverse behavioral effects in both fentanyl/midazolam sedation and ketamine/midazolam sedation; however, the prevalence was higher with fentanyl/midazolam sedation compared to ketamine/midazolam sedation, and the authors reported that these effects increased as the doses of fentanyl or ketamine increased [48]. Ritwik et al. reported increased irritability in children sedated with oral midazolam, reaching a prevalence of 32% compared to children sedated with oral meperidine and hydroxyzine (5%) in the first eight h after discharge than in the 8–24-h period; more children in the meperidine and hydroxyzine group became irritable, and less children in the midazolam group were irritable [35].

We also compared the behavior of children during their sedation visit in the first part of this clinical trial with the behavior of these children after discharge when they arrived home. Surprisingly, we found that more than half of the children who were calm during midazolam/fentanyl sedation became agitated at home, which is in agreement with McQueen et al.’s study [48], and most of the children who were anxious during sedation became calm at home, which could be because these children returned home exhausted after the unpleasant dental visit. Similarly, more than half of the children sedated with midazolam only who were agitated during dental treatment became calm at home. Conversely, more than half of children who were calm during midazolam-only sedation continued with calm behavior at home.

When the parents were asked about the sleeping pattern of their children, it was found that more than half of the children had normal sleep on the day of sedation for both sedation groups. More children with midazolam/fentanyl sedation had an increase in sleeping hours compared to those in the other group. This is in accordance with previous studies that found that using a combination of oral sedative drugs resulted in prolonged sleeping compared to oral midazolam-only sedation [33,35]. Costa et al. reported that high doses of oral midazolam sedation did not prolong sleeping in children as compared with children who received high doses of oral chloral hydrate [34].

More children in the midazolam/fentanyl group required a longer time to recover to normal cognitive function and balance compared to those in the other group, but this difference was not significant statistically. However, Kennedy et al. found that children receiving IV ketamine/midazolam had a longer recovery period compared to those receiving IV fentanyl/midazolam sedation [49]. Another study found that motor imbalance was more strongly associated with chloral hydrate sedation compared to midazolam sedation [32].

Moreover, more children in the midazolam/fentanyl group required a significantly longer time to start eating compared to those in the other group, which could be due to the deeper sedation of midazolam/fentanyl and the tendency to have a nap after the sedation, which could delay the meal time. However, Ritwik et al. reported a tendency of the children to not eat in the first eight h, regardless of the sedation regimen received [35]. In our study, the parents were requested through the post-discharge instructions to start with liquids and then with a light meal to maintain hydration of the child.

Parental perception of sedation is a vital factor in decision making at sedation clinics as the use of sedative drugs is dependent on parental consent for sedation and their perception of the degree of success of the sedation technique used to manage the child’s behavior [50]. However, only a few studies have been conducted that evaluate parents’ perceptions of sedation [51,52,53,54].

In this study, more parents were satisfied with midazolam/fentanyl sedation compared with midazolam-only sedation. When the parents were asked about the reason for their satisfaction regarding midazolam/fentanyl sedation, the responses indicated that it was because of the deeper sedation during dental treatment as perceived by the better and calmer behavior in the recovery room when they rejoined their child. Surprisingly, when the parents were asked after the second sedation visit about the preferred visit, more parents preferred midazolam-only sedation compared with midazolam/fentanyl sedation, and this was because of the faster recovery time. The parents reported that they worry and are more concerned about their children as they have to closely observe their child at home when the recovery time is long. This is in accordance with a previous study that found higher parent satisfaction with fast recovery from sedation [51]. By contrast, parents who preferred midazolam/fentanyl sedation indicated that this was because of the more cooperative and less anxious behavior of their children during the dental treatment. These parents reported that they were concerned about the psychological status of their child, and they did not like the visit where the child struggled and cried in the dental chair. This is in accordance with previous studies that found that parents’ satisfaction was more related to better sedated children with calm behavior [52,53,54]. However, some parents’ responses indicated no difference between the two sedation visits.

## 5. Conclusions

Intranasal fentanyl added to oral midazolam sedation could have an effect on post-discharge adverse behavioral changes, prolonged sleeping, and prolonged recovery time. Children sedated with midazolam/fentanyl required a longer amount of time until they ate their first post-sedation meal. Vomiting and fever occurred similarly in both sedation regimens, with a low incidence. There was no difference in parents’ preference regarding the two sedation regimens.

## Figures and Tables

**Table 1:** The demographic data of the children.

Number of children	32
Age (months)	54.6 ± 10.2
Gender (M/F)	18/14

**Table 2:** The different post-discharge adverse effects of the two sedation groups.

Variables	M/F		M		Wilcoxon *p*-Value
	Median	IQR	Median	IQR	
Vomiting frequency ^a^ Children who had a meal ^a^	2	0	2	0	0.564
	1	0	1	0	1
Time until first meal ^b^	3	1	2	1	0.04 *
Time until normal function ^b^	3	1	3	2	0.295
Time until normal balance ^b^	3	1	3	2	0.32
Behavior status ^c^	2	2	1.5	2	0.511
Sleeping pattern ^d^	1	1	1	1	0.755
Occurrence of side effects ^a^	2	0	2	0	1
Parent satisfaction ^e^	4	1.75	4	2	0.471

^a^: 1: yes, 2: no.; ^b^: 1: < 1 h, 2: 2–4 h, 3: 4–6 h, 4: > 6 h; ^c^: 1: normal, 2: more relaxed than usual, 3: more agitated/ aggressive than usual, 4: very agitated/aggressive; ^d^: 1: normal, 2: slept more than usual, 3: awake more than usual, 4: more nightmares than usual; ^e^: 1: very unsatisfied, 2: unsatisfied, 3: neutral, 4: satisfied, 5: very satisfied. * Statistically significant.

**Table 3:** The amount of time needed until the first meal after sedation for children in both groups.

Time	M/F No. of Children (%)	M No. of Children (%)
<1 h	0 (0%)	1 (3.2%)
2–4 h	13 (41.9%)	19 (61.3%)
4–6 h	11 (35.5%)	9 (29%)
>6 h	7 (22.6%)	2 (6.5%)
Total	32 (100%)	32 (100%)

## Data Availability

The datasets used and analyzed during the current study are available from the corresponding author upon reasonable request.

## Appendix A. Post-Sedation Phone-Call Verbal Questionnaire

1. **Did the patient vomit? (circle one)**
  Yes                              No
  **If yes, how much?.............................................................................................**
  **When?...............................................................................................................**
  **How soon after sedation?**
  <1 h      2–4 h      4–6 h      <6 h
2. **Did the patient eat? (circle one)**
  Yes                              No
  **How soon after sedation did your child eat?**
  <1 h      2–4 h      4–6 h      <6 h
3. **How long did it take for your child to function normally? (circle one)**
  <1 h      2–4 h      4–6 h      <6 h
4. **Were there any other side effects or complications?**
  ……….………………………………………………………………………………
5. **How would you rate your overall satisfaction with treatment using this medicine? (circle one)**
  (a) Very unsatisfied
  (b) Unsatisfied
  (c) Neutral
  (d) Satisfied
  (e) Very satisfied
6. **Which behavior best describes your child in the afternoon/evening following the sedation appointment?**
  (a) Normal
  (b) More relaxed than usual
  (c) More agitated/aggressive than usual
  (d) Very agitated/aggressive
7. **Please describe your child’s sleep after the sedation appointment.**
  (a) Normal
  (b) Slept more than usual
  (c) Awake more than usual
  (d) More nightmares than usual
8. **How many hours after the appointment before the child had normal balance and was able to walk normally? (circle one)**
  <1 h      2–4 h      4–6 h      <6 h
9. **How many hours after the appointment did your child experience normal vision? (no double or blurred vision) (circle one)**
  <1 h      2–4 h      4–6 h      <6 h
  **To be asked after the second sedation visit only:**
  **Did you prefer the medication given at the first appointment, second appointment, or no preference?**
  First visit      Second visit      Same/no preference

## References

[1] Erfanparast L., Vafaei A., Ranjkesh B., Sohrabi A., Bahadori Z., Pourkazemi M., Dadashi S., Shirazi S. (2015). Impact of Self-Concept on Preschoolers’ Dental Anxiety and Behavior. J. Dent. Res. Dent. Clin. Dent. Prospect..

[2] Swarna K., Prathima G., Suganya M., Sanguida A., Selvabalaji A. (2019). Recent Advances in Non-Pharmacological Behaviour Management Techniques in Children—An Overview. IOSR J. Dent. Med. Sci..

[3] Lourenço-Matharu L., Ashley P.F., Furness S. (2012). Sedation of Children Undergoing Dental Treatment. Cochrane Database Syst. Rev..

[4] American Academy of Pediatric Dentistry (2019). Reference Manual. Guidelines for Monitoring and Management of Pediatric Patients Before, During, and After Sedation for Diagnostic and Therapeutic Procedures: Update 2019. Pediatric Dent..

[5] American Dental Association Guidelines for the Use of Sedation and General Anesthesia by Dentists. https://www.ada.org/-/media/project/ada-organization/ada/ada-org/ada/ada/education-and-careers/files/ada_sedation_use_guidelines.pdf?rev=3dea7b89a7fb4a3a98eae26a76af493e&hash=A3A9CABCCDA5BB1D83E88F9146BD8997.

[6] Shapira J., Kupietzky A., Kadari A., Fuks A.B., Holan G. (2005). Comparison of Oral Midazolam with and Without Hydroxyzine in the Sedation of Pediatric Dental Patients. Pediatr. Dent..

[7] Torres-Pérez J., Tapia-García I., Rosales-Berber M.Á., Hernández-Sierra J.F., Pozos-Guillen A. (2007). Comparison of Three Conscious Sedation Regimens for Pediatric Dental Patients. J. Clin. Pediatr. Dent..

[8] Ahmadi R., Mozafar S., Bargrizan M., Golpayegani M.V., Shayeghi S. (2018). Comparison of Nitrous oxide/midazolam and Nitrous oxide/Promethazine for Pediatric Dental Sedation: A Randomized, Cross-Over, Clinical Trial. Dent. Res. J..

[9] Johnson E., Briskie D., Majewski R., Edwards S., Reynolds P. (2010). The Physiologic and Behavioral Effects of Oral and Intranasal Midazolam in Pediatric Dental Patients. Pediatr. Dent..

[10] Tavassoli-Hojjati S., Mehran M., Haghgoo R., Tohid-Rahbari M., Ahmadi R. (2014). Comparison of Oral and Buccal Midazolam for Pediatric Dental Sedation: A Randomized, Cross-Over, Clinical Trial for Efficacy, Acceptance and Safety. Iran. J. Pediatr..

[11] Gentz R., Casamassimo P., Amini H., Claman D., Smiley M. (2017). Safety and Efficacy of 3 Pediatric Midazolam Moderate Sedation Regimens. Anesth. Prog..

[12] Papineni A., Lourenço-Matharu L., Ashley P. (2012). Safety of Oral Midazolam Sedation Use in Paediatric Dentistry: A Review. Int. J. Paediatr. Dent..

[13] Kılıç E.T., Akcay M.E., Akdemir M.S. (2018). The Comparison of the Efficacy and Safety of Midazolam, Ketamine, and Midazolam Combined with Ketamine Administered Nasally for Premedication in Children. Anesth. Essays Res..

[14] Payne K., Mattheyse F.J., Liebenberg D., Dawes T. (1989). The Pharmacokinetics of Midazolam in Paediatric Patients. Eur. J. Clin. Pharmacol..

[15] Reed M.D., Rodarte A., Blumer J.L., Khoo K.-C., Akbari B., Pou S., Kearns G.L. (2001). The Single-Dose Pharmacokinetics of Midazolam and Its Primary Metabolite in Pediatric Patients After Oral and Intravenous Administration. J. Clin. Pharmacol..

[16] Sado-Filho J., Viana K.A., Corrêa-Faria P., Costa L.R., Costa P.S. (2019). Randomized Clinical Trial on the Efficacy of Intranasal or Oral Ketamine-Midazolam Combinations Compared to Oral Midazolam for Outpatient Pediatric Sedation. PLoS ONE.

[17] Manso M.A., Guittet C., Vandenhende F., Granier L. (2019). Efficacy of Oral Midazolam for Minimal and Moderate Sedation in Pediatric Patients: A Systematic Review. Pediatr. Anesth..

[18] Ryan P.M., Kienstra A.J., Cosgrove P., Vezzetti R., Wilkinson M. (2019). Safety and Effectiveness of Intranasal Midazolam and Fentanyl Used in Combination in the Pediatric Emergency Department. Am. J. Emerg. Med..

[19] Murphy A.P., Hughes M., Mccoy S., Crispino G., Wakai A., O’Sullivan R. (2017). Intranasal Fentanyl for the Prehospital Man-Agement of Acute Pain in Children. Eur. J. Emerg. Med..

[20] Nemeth M., Jacobsen N., Bantel C., Fieler M., Sümpelmann R., Eich C. (2019). Intranasal Analgesia and Sedation in Pediatric Emergency Care-A Prospective Observational Study on the Implementation of an Institutional Protocol in a Tertiary Children’s Hospital. Pediatr. Emerg. Care..

[21] Pansini V., Curatola A., Gatto A., Lazzareschi I., Ruggiero A., Chiaretti A. (2021). Intranasal Drugs for Analgesia and Sedation in Children Admitted to Pediatric Emergency Department: A Narrative Review. Ann. Transl. Med..

[22] Streisand J.B., Zhang J., Niu S., McJames S., Natte R., Pace N.L. (1995). MD Buccal Absorption of Fentanyl Is PH-Dependent in Dogs. Anesthesiol. J. Am. Soc. Anesthesiol..

[23] Mamula P., Markowitz J.E., Neiswender K., Zimmerman A., Wood S., Garofolo M., Nieberle M., Trautwein A., Lombardi S., Sargent-Harkins L. (2007). Safety of Intravenous Midazolam and Fentanyl for Pediatric GI Endoscopy: Prospective Study of 1578 Endoscopies. Gastrointest. Endosc..

[24] Lee B., Park J.D., Choi Y.H., Han Y.J., Suh D.I. (2019). Efficacy and Safety of Fentanyl in Combination with Midazolam in Children on Mechanical Ventilation. J. Korean Med Sci..

[25] Williams M.R., Nayshtut M., Hoefnagel A., McKeown A., Carlson D.W., Cravero J., Lightdale J., Mason K.P., Wilson S., Turk D.C. (2018). Efficacy Outcome Measures for Pediatric Procedural Sedation Clinical Trials. Anesth. Analg..

[26] Klein E.J., Diekema D.S., Paris C.A., Quan L., Cohen M., Seidel K.D. (2002). A Randomized, Clinical Trial of Oral Midazolam Plus Placebo Versus Oral Midazolam Plus Oral Transmucosal Fentanyl for Sedation During Laceration Repair. Pediatrics.

[27] Pandey R.K., Padmanabhan M.Y., Saksena A.K., Chandra G. (2010). Midazolam-Fentanyl Analgo-Sedation in Pediatric Dental Patients—A Pilot Study. J. Clin. Pediatr. Dent..

[28] Oh S., Kingsley K. (2018). Efficacy of Ketamine in Pediatric Sedation Dentistry: A Systematic Review. Compend. Contin. Educ. Dent..

[29] Mohite V., Baliga S., Thosar N., Rathi N. (2019). Role of Dexmedetomidine in Pediatric Dental Sedation. J. Dent. Anesthesia Pain Med..

[30] Practice Guidelines for Moderate Procedural Sedation and Analgesia 2018: A Report by the American Society of Anesthesiologists Task Force on Moderate Procedural Sedation and Analgesia, the American Association of Oral and Maxillofacial Surgeons, American College of Radiology, American Dental Association, American Society of Dentist Anesthesiologists, and Society of Interventional Radiology. https://pubs.asahq.org/anesthesiology/article/128/3/437/18818/Practice-Guidelines-for-Moderate-Procedural.

[31] Coteé C.J., Karl H.W., Notterman D.A., Weinberg J.A., McCloskey C. (2000). Adverse Sedation Events in Pediatrics: Analysis of Medications Used for Sedation. Pediatrics.

[32] Malviya S., Voepel-Lewis T., Prochaska G., Tait A.R. (2000). Prolonged Recovery and Delayed Side Effects of Sedation for Diagnostic Imaging Studies in Children. Pediatrics.

[33] Martinez D., Wilson S. (2006). Children Sedated for Dental Care: A Pilot Study of the 24-Hour Postsedation Period. Pediatrics Dent..

[34] Costa L.R., Costa P.S., Brasileiro S.V., Bendo C.B., Viegas C.M., Paiva S.M. (2012). Post-Discharge Adverse Events Following Pediatric Sedation with High Doses of Oral Medication. J. Pediatrics.

[35] Ritwik P., Cao L.T., Curran R., Musselman R.J. (2013). Post-Sedation Events in Children Sedated for Dental Care. Anesth. Prog..

[36] McCormack L., Chen J.-W., Trapp L., Job A. (2014). A Comparison of Sedation-Related Events for Two Multiagent Oral Sedation Regimens in Pediatric Dental Patients. Pediatrics Dent..

[37] Huang A., Tanbonliong T. (2015). Oral Sedation Postdischarge Adverse Events in Pediatric Dental Patients. Anesth. Prog..

[38] American Society of Anesthesiologist Physical Status Classification System. https://www.asahq.org/standards-and-guidelines/asa-physical-status-classification-system.

[39] Frankl S.N. (1962). Should the Parent Remain with the Child in the Dental operatory?. J. Dent. Child.

[40] Mallampati S.R., Gatt S.P., Gugino L.D., Sukumar P.D., Waraksa B., Freiberger D., Liu P.L. (1985). A Clinical Sign to Predict Difficult Tracheal Intubation; A Prospective Study. Can. Anaesth. Soc. J..

[41] Brodsky L. (1989). Modern Assessment of Tonsils and Adenoids. Pediatrics Clin. North Am..

[42] El-Mouzan M.I., Al-Herbish A.S., Al-Salloum A.A., Qurachi M.M., Al-Omar A.A. (2007). Growth Charts for Saudi Children and Adolescents. Saudi Med. J..

[43] Sheta S.A., AlSarheed M. (2009). Oral Midazolam Premedication for Children Undergoing General Anaesthesia for Dental Care. Int. J. Pediatrics.

[44] MOH Extemporaneous Formulary Pharmaceutical Services Division Ministry of Health Malaysia. https://www.pharmacy.gov.my/v2/sites/default/files/document-upload/moh-extemporaneous-formulary-2011.pdf.

[45] Nahata M., Pai V., Hipple T. (2011). Pediatric Drug Formulation.

[46] Rech M.A., Barbas B., Chaney W., Greenhalgh E., Turck C. (2017). When to Pick the Nose: Out-of-Hospital and Emergency Department Intranasal Administration of Medications. Ann. Emerg. Med..

[47] American Academy of Pediatric Dentistry Reference Manual, Preparing for Your Child’s Sedation Visit. https://www.aapd.org/globalassets/media/policies_guidelines/r_prepostsedation.pdf.

[48] McQueen A., Wright R.O., Kido M.M., Kaye E., Krauss B. (2009). Procedural Sedation and Analgesia Outcomes in Children After Discharge from the Emergency Department: Ketamine Versus fentanyl/midazolam. Ann. Emerg. Med..

[49] Kennedy R.M., Porter F.L., Miller J.P., Jaffe D.M. (1998). Comparison of Fentanyl/Midazolam with Ketamine/Midazolam for Pediatric Orthopedic Emergencies. Pediatrics.

[50] Hagan P.P., Hagan J.P., Fields H.W., Machen J.B. (1984). The Legal Status of Informed Consent for Behavior Management Techniques in Pediatric Dentistry. Pediatrics Dent..

[51] Kip G., Atabek D., Bani M. (2018). Comparison of Three Different Ketofol Proportions in Children Undergoing Dental Treatment. Niger. J. Clin. Pr..

[52] Lima A.R.D.A., Medeiros M., Costa L.R. (2015). Mothers’ Perceptions about Pediatric Dental Sedation as an Alternative to Dental General Anesthesia. RGO.

[53] Rodrigues V.B.M., Costa L.R., de Faria P.C. (2021). Parents’ Satisfaction with Paediatric Dental Treatment under Sedation: A cross-sectional Study. Int. J. Paediatr. Dent..

[54] Lew V.K., Lalwani K., Palermo T.M. (2010). Factors Affecting Parental Satisfaction Following Pediatric Procedural Sedation. J. Clin. Anesth..

